# Neonatal Abandonment and Hydrocephalus in Antillean Manatees (*Trichechus manatus manatus*): Is There a Causal Relationship?

**DOI:** 10.3390/ani15020161

**Published:** 2025-01-10

**Authors:** Tiago F. S. Santos, Márcio A. O. Moura, Gabriel S. F. Tavares, Julyanne S. Siqueira, Natália M. F. P. Sarmento, Robert G. S. Prado, Alexandra F. Costa, Tayanna M. M. M. Amaral, Renata Emin-Lima, Maura E. M. Sousa, Reginaldo H. M. Moreira Júnior, Pedro S. Bezerra Júnior, Gabriela Riet-Correa, Valíria D. Cerqueira

**Affiliations:** 1 Animal Pathology Laboratory (LAPATO), Institute of Veterinary Medicine (IMV), Federal University of Pará (UFPA), Castanhal 68745-000, PA, Brazil; mmarcioalan@yahoo.com.br (M.A.O.M.); nataliasarmento7@gmail.com (N.M.F.P.S.); pradosanch5@gmail.com (R.G.S.P.); valiria@ufpa.br (V.D.C.); 2 Bicho D’água Institute: Socio-Environmental Conservation (IBD), Soure 68870-000, PA, Brazil; 3 Chico Mendes Institute for Biodiversity Conservation (ICMBio), Coral Coast Environmental Protection Area (APACC), Tamandaré 55578-000, PE, Brazil

**Keywords:** marine mammals, Amazon, abandonment, congenital pathology

## Abstract

**Simple Summary:**

Antillean manatees are among the most endangered marine animals, and there is a paucity of data on free-living animals’ behavior in the northern region of Brazil, where they occur in sympatry with the Amazonian manatee. This study highlights the interaction behavior of adult manatees with a calf that was unable to remain stable in the water and was carried away by the river tide. The neonate was later rescued and died, and it was possible to diagnose congenital hydrocephalus (an increase in cerebrospinal fluid in the brain), the main factor that may have influenced the stranding and possible abandonment of the neonate by the adult animals, since the condition causes difficulties in locomotion and feeding, and may be a disadvantage for the survival of the neonate and the adults.

**Abstract:**

Manatees are semi-social animals, with the mother–calf relationship being considered long-lasting for the species. However, some events lead to the separation of this pair. Orphaned manatee calves can be adopted by other females of the same species. However, if this does not happen and a healthy calf strand is rescued, immediate release represents the best option for the individual. But when immediate release becomes unviable, the animals are taken to rehabilitation centers and can die from various causes. A newborn Antillean manatee was rescued in the north of Brazil, and the attempt at immediate release was unsuccessful; three months later, the animal died. At necropsy, it was observed that the brain was soft and friable, collapsing when placed on a surface, with the corpus callosum region able to be ruptured easily and the cerebral hemispheres lying flaccid. The analysis of serial sections of the brain showed dilated lateral ventricles and a reduction in white matter, making it possible to affirm the presence of congenital hydrocephalus, the main factor that may have led to the abandonment of the offspring.

## 1. Introduction

Manatees (*Trichechus* spp.) are semi-social animals and are occasionally found in small groups which generally split up quickly. The intraspecific relations of these animals have low stability, with the mother–calf relationship being one of the few that is considered long-lasting for the species, with this pair remaining together for around two years [1,2].

These animals have pregnancies that last 12 to 14 months, usually giving birth to a single neonate. In the first few hours after birth, the offspring is helped by its mother to swim, to emerge, and to breathe [2,3,4,5].

Sirenians dedicate a lot of time and energy to long-term parental care behavior, in which the mother stays with the calf to feed it, protect it, and teach it about displacement patterns, feeding sites, sources of fresh water, and possible dangers to increase the offspring’s chances of survival. Additionally, females have been seen carrying a dead neonate for days (epimeletic behavior), reinforcing the maternal nature of the species [4,6]. 

This mother–offspring connection allows for the development of matrilineal behavior and natal philopatry, in which animals, especially females, continue to use the same areas in which they were born, with evidence of manatees from three generations using the same area [7].

Although they have this strong mother–calf relationship, some events lead to the separation of this pair, including the growth of anthropogenic activities due to actions such as the obstruction and destruction of habitats used as nurseries, inducing the female to give birth on the open sea, leaving the offspring exposed to sea currents, which can cause the separation of mother and offspring [8,9].

In the Amazon region, even though this practice is prohibited by law, manatees are still hunted for subsistence by riverside populations [10,11], and the slaughter of the mother is a factor that can contribute to the stranding of healthy calves. Orphaned manatee calves can be adopted by other females of the same species (allomaternal care behavior), a process that has already been described in sirenians in situ (free-living) and ex situ (under human care) [12,13].

Antillean manatees are categorized globally as “vulnerable” to extinction and nationally as “in danger” of extinction [14,15]. These classifications may be caused by the current threats to the species and its distribution, such as environmental degradation influenced by anthropogenic activities, as mentioned above, and, historically, by intentional capture, leading to drastic population reductions in some areas of its distribution, leading to the creation of laws to protect the species [15]. 

Once a healthy calf strands and is rescued, immediate release represents the best option for the individual, since there is no need for a rehabilitation process, requiring only the stabilization of the animal, observation of the presence of adult animals in the area, favorable environmental conditions, and the possibility of temporary monitoring in the region [16].

However, when immediate release becomes unviable, the animals are taken to rehabilitation centers, where they can be released at the end of a long process [17], which is not always completed, with animals dying from various causes, such as congenital, traumatic, bacterial, parasitic, fungal, and viral pathologies [18].

Hydrocephalus is one of the pathologies already diagnosed in Antillean manatees calf undergoing rehabilitation [19]. It is characterized by the abnormal accumulation of cerebrospinal fluid in the ventricular system of the CNS and can be congenital or acquired [20].

With the aim of contributing to data on the species’ intraspecific behavior with animals presenting congenital pathologies, this paper reports a possible neonatal rejection of Antillean manatees (*Trichechus manatus manatus*) presenting congenital hydrocephalus.

## 2. Materials and Methods

**Figure 1:**
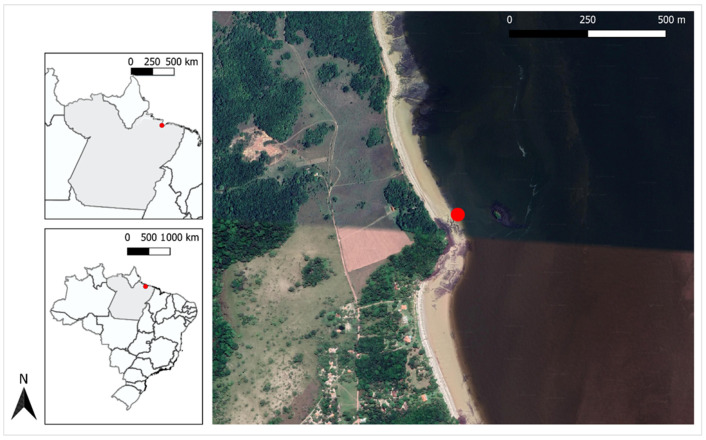
Stranding site where the Antillean manatee calf (*Trichechus manatus manatus*) was found (red dot).

An Antillean manatee newborn (*T. m. manatus*) was rescued by fishermen on the east coast of the Marajó archipelago (0.830065 S, 48.513772 O) in northern Brazil (Figure 1); the fishermen notified local environmental agencies and researchers from the Bicho D’água Institute (IBD). 

Initially, an immediate release attempt was made by local collaborators (who had previously been trained by the IBD team), who confirmed the presence of adult animals in the region; however, after the release, it was noticed that the calf had difficulties with the river tide, and it was rescued a second time and taken to a place where there was another manatee in the process of rehabilitation, in the same municipality, for initial care.

In this second rescue, the IBD team was at the rehabilitation site, where physical examinations were carried out (external inspection and inspection of the oral, ocular, umbilical, anal, and genital mucous membranes, as well as cardiac and pulmonary auscultation and biometrics), followed by observation of their behavior in and out of the pool and feeding with soy milk replacer. At the same time, attempts were made to locate adult animals in the area in order to try to release the calf again.

**Figure 2:**
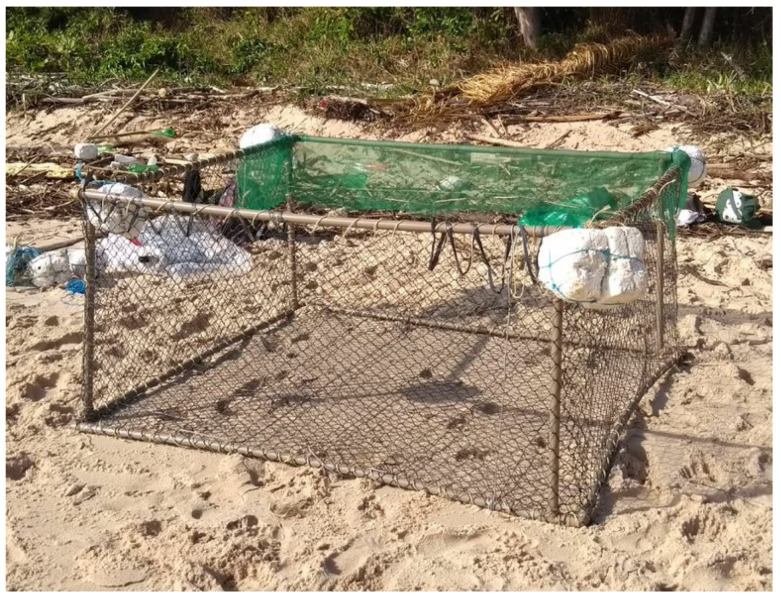
Floating enclosure built to help the cub get closer to the adults in the area.

On the second release attempt, the animal was taken to the same area as the previous stranding, where a floating enclosure was improvised (Figure 2) using fishing nets, pipes, and buoys. The calf was placed in the enclosure to bring it safely closer to the adult animals in the area, and the release was monitored using a boat without an engine and at a fixed point. However, the attempt was also unsuccessful.

Two attempts at immediate release were unsuccessful, and the cub was sent back to the rehabilitation area, where it remained under the observation and care of the IBD team.

After three months of care, the animal died and was sent to the Animal Pathology Laboratory (LAPATO) of the Federal University of Pará (UFPA) for necroscopic examination, which was carried out according to the protocol of Bonde et al. (1983) [21].

Organ fragments were collected during necropsy, fixed in 10% buffered formalin, and routinely processed for histopathological analysis, with the organ fragments being embedded in paraffin and cut with a microtome (5 µm) and then stained with hematoxylin and eosin (H&E) [22]. In addition, serial coronal sections of the formalin-fixed brain (~1.5 cm) were made for macroscopic observation and anatomopathological evaluation.

## 3. Results

**Figure 3:**
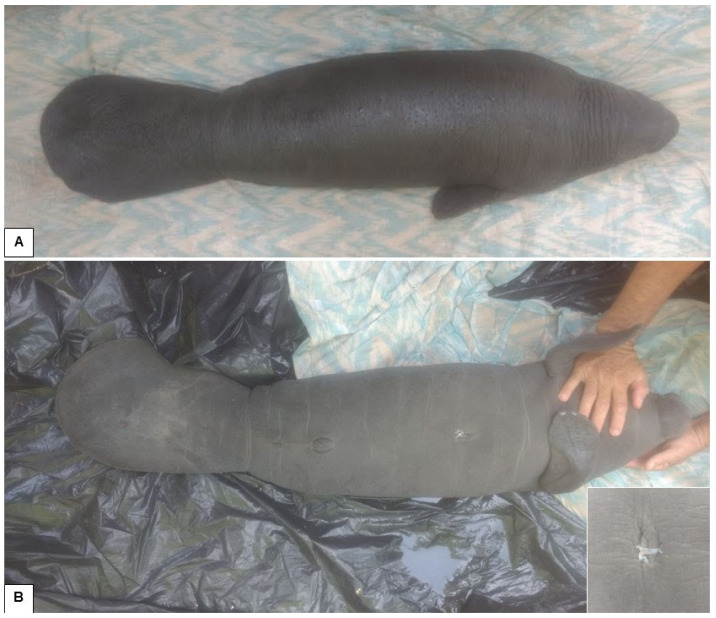
A female Antillean manatee at the stranding site. (**A**) Dorsal view. (**B**) Ventral view, showing the presence of the nails, characteristic of the American manatee (*Trichechus manatus*). Magnified photo detailing the umbilical cord that was still present.

The female Antillean manatee was rescued with remnants of the umbilical cord (Figure 3). The clinical examination showed no changes in mucous membranes, heart rate, respiratory rate, or behavior. She was also feeding normally on the formula offered.

**Figure 4:**
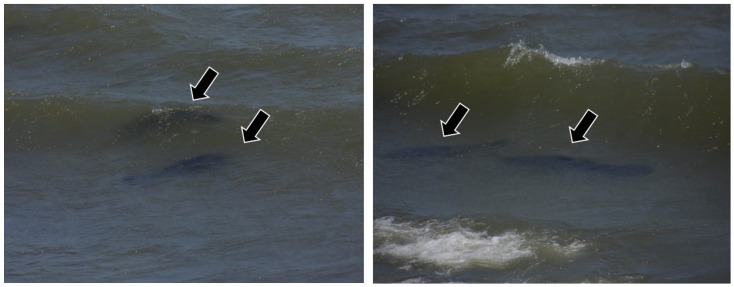
Presence of two adult manatees (arrow) near the area where the calf stranded.

During the search, two adult animals were found near the stranding area (Figure 4), one of which was thought to be the mother. Due to the presence of adults, the absence of clinical signs and favorable environmental conditions, an attempt was made to release the calf immediately by local collaborators, but this was unsuccessful, possibly due to the strong river tide, which the calf was unable to withstand, getting tired and being carried away by the current. However, the adult animals were seen approaching the calf, but they did not make physical contact.

On the second release attempt, the offspring was placed in the water inside the floating enclosure, where she vocalized frequently. The enclosure was brought close to the adult animals by a person in the water and monitored from afar by the team, both on board and at a fixed point on land. When approaching the adults, it was possible to hear their vocalizations underwater, as well as those of the calf. Near the adults, the enclosure was removed, leaving the calf free. However, similar to the previous attempt, the adult animals approached but did not make physical contact with the calf, and as time went by, the calf became tired and was carried away by the river tide. 

Approximately 45 min after the animal had been in the water and its behavior observed, it was decided to rescue it again and take it to the rehabilitation center. Over the days and as the animal was monitored at the rehabilitation center, it was observed that she was exhibiting atypical behaviors, showing lateralization to the left, with difficulty in diving to the bottom of the tank, always swimming in an anti-clockwise direction, and repeatedly crashing into the wall of the tank.

**Figure 5:**
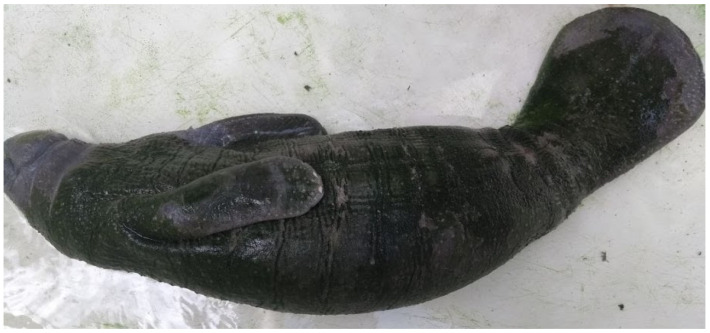
Image showing the presence of a large amount of algae on the calf’s skin.

Three months later, in the rehabilitation tank, the animal had not progressed clinically, showing feeding difficulties, apathy, and high algae proliferation (Figure 5). Eventually, it died, and it was then sent to LAPATO/UFPA for necropsy.

**Figure 6:**
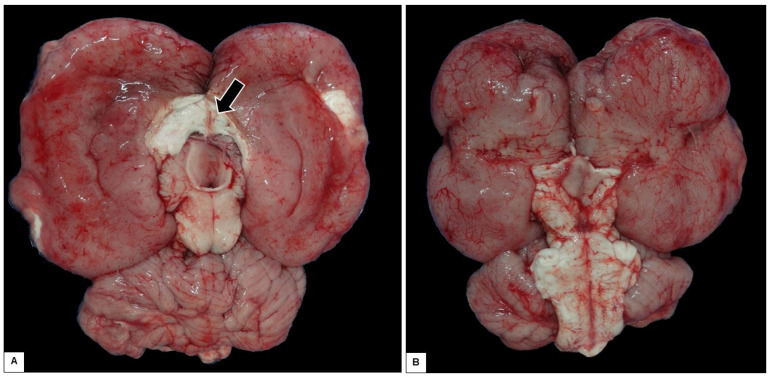
Gross appearance of the encephalon with hydrocephalus. It collapsed after removal of the skullcap, leading to the rupture of the corpus callosum (arrow). (**A**) Dorsal view. (**B**) Ventral view.

**Figure 7:**
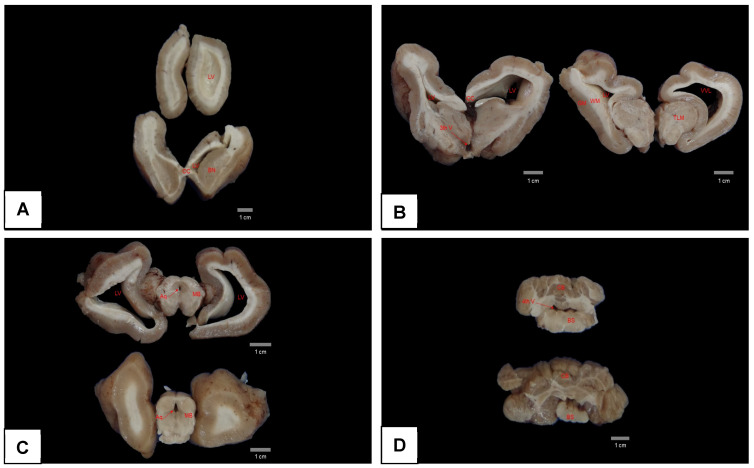
Serial coronal sections of the brain of an Antillean manatee with hydrocephalus fixed in formalin. Note in (**A**–**C**) an enlargement of the lateral ventricles and a reduction in white matter, mainly in right side. No significant changes were observed in figure (**D**). LV = lateral ventricle; CC = corpus callosum; BN = basal nuclei; 3th V = third ventricle; GM = grey matter; WM = white matter; TLM = thalamus; Aq = aqueduct; MB = midbrain CB = cerebellum; 4th V = fourth ventricle; BS = brainstem.

At necropsy, pulmonary congestion, edema, and ulcers in the duodenum were observed. However, the most important finding was encountered after opening the skullcap, where it was observed that the brain was soft and friable, collapsing when placed on a surface, with the corpus callosum region able to be ruptured easily and the cerebral hemispheres lying flaccid (Figure 6). Analysis of serial sections of the brain showed dilated lateral ventricles, atrophy of the hippocampus, and thinning of the white matter (Figure 7A–D). 

Histopathology showed white matter thinning, mainly in the cerebral cortex near the lateral ventricles, with vacuolization of the neuroepithelium in the deep layer of the grey matter and in the white matter. In the same areas, the ependymal cells were flattened. Another finding of no clinical importance was focal and discrete perivascular mononuclear cell cuffs in the white matter of the cerebral cortex.

## 4. Discussion

Records of manatee strandings in Brazil, especially that of young ones, remain constant, with occasional increases in cases over the years, mainly in the northeast [23], which may be associated with the fact that, in this area, there is a stronger stranding network, as well as beach monitoring projects and easier access to places where animals strand.

The geographical features of the north of Brazil, due to its size and characteristics associated with locomotion, which is largely carried out by rivers, as well as the presence of large tides, make it difficult to access some locations, making it impossible for the stranding network to attend to many cases of stranded animals, which means that the number of records is much lower than the real number.

Stranding events offer opportunities to study the health, ecology, and behavior of marine mammal species [24], and it is particularly important when it comes to species such as manatees, which exhibit furtive behavior and a high lung capacity, as they can remain submerged for many minutes [25], making it difficult to observe them in their natural habitat. Furthermore, places with characteristics that make it difficult to study free-living marine mammals, such as the Marajó Bay region, which has turbid waters and low visibility [26], also make it difficult to obtain information about these species.

According to Bonvicino et al. (2020) [27], between 2005 and 2018, 35 manatee strandings were recorded in the state of Pará, 16 of which occurred on the Marajó coast, and 6 of which involved Antillean manatees, animals that, until 2005, were believed to be extinct in the region due to predatory hunting [28,29]. 

It is now known that Marajó Bay is one of the sites of sympatry for two species of manatee, Antillean manatee (*Trichechus manatus manatus*) and Amazonian manatee (*Trichechus inunguis*) [30], making strandings in this region even more valuable for studying these populations and reinforcing the importance of the region for the conservation of sirenian species found in Brazil.

The attempt to release the newborn manatee immediately represented the best chance of survival for the animal, which had a favorable evaluation for release, as it showed no clinical alterations during handling, in addition to the presence of adult individuals in the area and environmental conditions that did not pose a danger to the animal, as proposed by Attademo et al. (2022) [16].

Although much remains to be studied about the behavioral patterns of manatees, especially in free-living animals, Attademo et al. (2020) [31] described various behavioral patterns, including some that represent clinical alterations, which must be associated with other variables.

The leftward lean presented by the animal at the rehabilitation center led to suspicions of a compromised respiratory or digestive system [31]. Due to the difficulty she experienced in diving to the bottom of the pool, there was also a suspicion that the animal’s digestive system was compromised. However, these suspicions were excluded at the necropsy, so there was a high possibility that these alterations were related to changes in the central nervous system (CNS).

The behavioral abnormalities described in this study corroborate changes in the animal’s CNS, and a similar situation was reported by Carvalho et al. (2019) [19], who diagnosed hydrocephalus in a young Antillean manatee which showed behavioral changes, staying for long periods with its belly up and pressing its head against the bottom of the pool.

Congenital alterations in Sirenia are rare, with few cases reported in the literature [19,32,33,34,35,36]. These alterations may be related to low genetic variability in free-living populations or, in the case of hydrocephalus, intrauterine infections, as happens in domestic animal species [20,37], which may have happened to the animal in this report, which showed a discrete lymphoplasmocytic inflammatory infiltrate around the vessels of the encephalon. But, we consider this to be an incidental finding, similar to what has been described and well documented in cattle [38].

Sirenia have moderate visual acuity and small optic nerves, and the cerebral cortex related to vision is present in the dorsal and dorsolateral portions of the brain [39,40], which, based on this study, are thought to be affected by the increased intracranial pressure caused by hydrocephalus, leading to a loss of brain tissue [20], causing reduced visual acuity or even cortical blindness, justifying the animal’s shocks against the tank walls.

Low visual acuity and central blindness have been reported in animals and humans and have already been associated with hydrocephalus, with this pathology causing lesions in the optic nerve or cortex related to vision [41,42,43].

**Figure 8:**
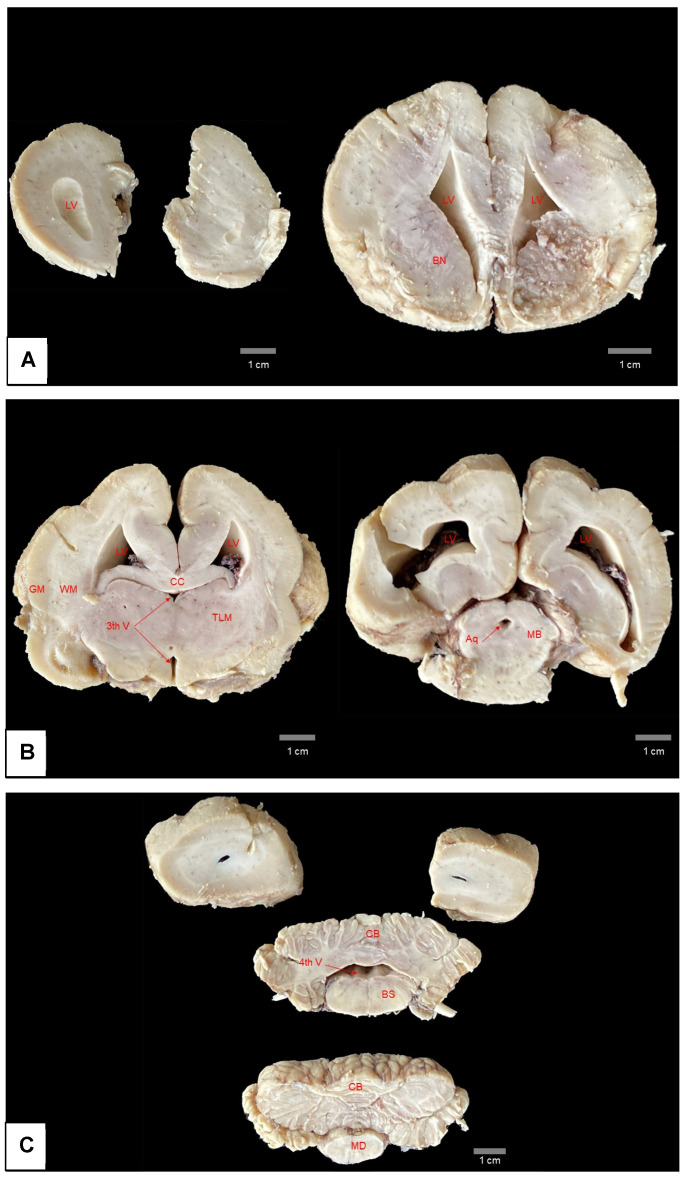
Serial section of brain of Antillean manatee without pathological alterations. The brain sections were fixed in formaldehyde. Note in (**A**–**C**) the white and gray matter of the encephalon with normal thickness and the dilation of the lateral ventricles, normally observed for the species. LV = lateral ventricle; BN = basal nuclei; CC = corpus callosum; 3th V = third ventricle; GM = grey matter; WM = white matter; TLM = thalamus; Aq = aqueduct; MB = midbrain; CB = cerebellum; 4th V = fourth ventricle; BS = brainstem; MD = medulla. Photo: LAPATO/UFPA.

Diagnosing hydrocephalus in manatees which do not show marked macroscopic alterations and have no visible cause, as is the case in other animal species [20], can be difficult to perform with certainty, as the species has enlarged lateral ventricles and few circumvolutions (lysencephalic encephalon) (Figure 8) [44,45], characteristics used in domestic animal species as part of the criteria for diagnosing hydrocephalus.

Despite this, the spontaneous rupture in the region of the corpus callosum when the brain was placed on a hard surface, after opening the skullcap, indicates a thinning of the nerve tissue, probably caused by the increased pressure and dilation of the lateral ventricle caused by hydrocephalus, (which is noticeable when comparing images of the present case (Figure 6) and the brain of an animal with no anatomical alterations (Figure 8).

Macroscopic evaluations of the CNS of manatees in Brazil only occur on a few occasions, such as when there are clinical signs that strongly suggest brain involvement, and as a result, there are few current and detailed parameters of the brain anatomy of these animals [44,45], which can make it difficult to diagnose pathologies such as hydrocephalus.

The present study hypothesizes that the female Antillean manatee and one of the wild animals present during the release had a parental relationship, considering the matrilineal behavior and natal philopatry displayed by the species, associated with the closeness of the animals to the calf. However, due to the inability to collect samples from free-living animals, we are unable to confirm this theory.

However, even if this hypothesis is not true and the wild animals were not directly related to the female Antillean manatee, it was clear that these animals were interested in the calf during both release attempts, which could have resulted in the calf being adopted, as has been described on other occasions [12,13], a situation that did not occur in this case.

Regardless of the hypothesis, it was clear that the animals present in the release area had abandoned the neonate, probably as a result of the congenital hydrocephalus that caused behavioral changes in the female Antillean manatee, which would be detrimental to the survival of the other animals.

## 5. Conclusions

Despite the anatomical particularities of the manatee encephalon, it is possible to affirm, in this case, the presence of congenital hydrocephalus, a diagnosis associated with the manatee calf’s swimming difficulties and low visual acuity, clinical signs that suggest the presence of alterations in the central nervous system. In addition, upon macroscopic examination of the brain, it was found to be friable and softened, findings related to an increase in cerebrospinal fluid in the ventricular system.

The hydrocephalus recorded in the offspring is the main aggravating factor found in the study, which could explain the abandonment by free-living animals in the release area. Knowing the compromises to the performance of some behaviors considered normal for the species, such as diving when fleeing and even feeding, this could pose risks to the survival of the animals involved.

Additionally, this case highlights the importance of opening the skull in animal necropsies so that the brain can be assessed, even in animals that have not shown clinical signs related to the CNS. This procedure aids diagnosis and generates more parameters for comparison on the particularities of the manatee encephalon.

## Data Availability

Data are contained within the article.
